# Mechanical versus Biological Valve Prostheses for Infective Endocarditis Presenting with Stroke

**DOI:** 10.3390/jcm13195712

**Published:** 2024-09-25

**Authors:** Amila Kahrovic, Philipp Angleitner, Harald Herkner, Paul Werner, Alexandra Andreeva, Thomas Poschner, Severin Laengle, Alfred Kocher, Guenther Laufer, Martin Andreas

**Affiliations:** 1 Department of Cardiac Surgery, Medical University of Vienna, 1090 Vienna, Austria; 2 Department of Emergency Medicine, Medical University of Vienna, 1090 Vienna, Austria

**Keywords:** infective endocarditis, stroke, biological valve prosthesis, mechanical valve prosthesis, neurological deterioration

## Abstract

**Objectives:** This study aimed to compare the clinical outcomes of mechanical and biological valve prostheses in patients with infective endocarditis presenting with stroke. **Methods:** Ninety-five adults with infective endocarditis complicated by stroke at baseline who underwent aortic and/or mitral valve replacement were analyzed retrospectively. The primary outcome was a composite outcome of all-cause mortality, ischemic stroke, hemorrhagic stroke, and re-endocarditis. Secondary outcomes included the individual components of the composite outcome and modified Rankin scale deterioration during follow-up. **Results:** Among the study cohort, 34 patients (35.8%) received mechanical valve prostheses and 61 (64.2%) received biological valve prostheses. Implantation of a mechanical valve prosthesis seems to be associated with a decreased risk of attaining the composite outcome (adjusted HR 0.46, 95% CI 0.22–0.96, and *p* = 0.037). Analyses of the individual components of the composite outcome showed that implantation of a mechanical valve prosthesis might not be associated with an increased risk of ischemic stroke, hemorrhagic stroke, and all-cause mortality during the follow-up period. Further, the risk of re-endocarditis was significantly lower in recipients of a mechanical valve prosthesis (adjusted HR 0.15, 95% CI 0.06–0.77, *p* = 0.026). Notably, a trend toward decreased risk of modified Rankin scale deterioration throughout the follow-up period was observed in this group (adjusted odds ratio 0.22, 95% CI 0.05–1.02, *p* = 0.053). **Conclusions:** Implantation of mechanical valve prostheses in patients presenting with infective endocarditis complicated by stroke seems to be beneficial in terms of a reduced risk of experiencing a composite outcome. Analyses of larger cohorts are required to validate our findings.

## 1. Introduction

Infective endocarditis (IE) frequently presents as a stroke, with an incidence ranging from 20% to 40% [1,2]. As of now, no published data are available regarding long-term clinical outcomes after the implantation of mechanical or biological valve prostheses in patients presenting with IE complicated by stroke. Indeed, endocarditis guidelines are inconclusive regarding the precise indications of prosthesis selection and long-term outcomes in this subset of patients [3,4,5].

The potential downside of implanting a mechanical valve prosthesis in patients presenting with stroke might be stroke exacerbation related to mandatory anticoagulant therapy [6,7]. Conversely, anticoagulant therapy might provide protection against new stroke onsets during the follow-up period and potentially enhance long-term neurological outcomes [8,9,10,11].

The implantation of a biological valve prosthesis may mitigate concerns related to hemorrhagic conversion of the infracted cerebral tissue [4]. However, it is important to consider that younger patients still face the inherent risk of structural valve deterioration and the potential need for reoperation [12].

In the setting of IE, it is plausible that the implantation of a mechanical valve prosthesis, which involves using more artificial material within the infected and inflamed environment, might be associated with an increased risk of re-endocarditis.

We aimed to retrospectively analyze the clinical outcomes in recipients of mechanical versus biological valve prostheses for IE complicated by stroke.

## 2. Materials and Methods

### 2.1. Ethical Statement

The Ethics Committee of the Medical University Vienna (vote number 1210/2022; approval date: 5 April 2022) granted approval for this study, and the need for individual patient consent was waived.

### 2.2. Patients and Clinical Data

Patient screening was conducted by reviewing the regularly updated database of the Department of Cardiac Surgery, Medical University of Vienna, between January 2009 and December 2020. A total of 95 consecutive patients with IE complicated by ischemic or/and hemorrhagic stroke within 2 months prior to surgery were included in this retrospective study. The modified Duke criteria were used to diagnose IE [13]. Based on the Stroke Council of the American Heart Association/American Stroke Association, stroke was defined as an acute neurological impairment accompanied by positive findings on computed tomography and/or magnetic resonance imaging [14]. All patients underwent surgical aortic and/or mitral valve replacement with either mechanical or biological valve prostheses. Patients beyond 18 years of age, as well as those who underwent isolated mitral valve repair and implantation of a homograft, were excluded. Additionally, patients with missing preoperative neuroimaging confirmation of stroke as well as those with isolated meningoencephalitis, intracerebral abscess, infectious intracranial aneurysm, and encephalopathy were excluded. Patients were grouped based on the type of valve prosthesis implanted (mechanical vs. biological valve prostheses).

### 2.3. Study Outcomes

The primary study outcome was a composite outcome of all-cause mortality, ischemic stroke, hemorrhagic stroke, and re-endocarditis. Secondary study outcomes included individual components of the composite outcome and modified Rankin scale (mRS) deterioration during follow-up. Concerning long-term neurological impairment, the mRS was used to assess the degree of disability in activities of daily living [15]. The mRS score was evaluated at baseline and follow-up, with an mRS score of ≥ 4 denoting severe disability or death. The mRS deterioration was defined as the worsening of a neurological deficit compared to the degree of disability prior to surgery. Study outcomes were extracted retrospectively from patients’ medical records. Mortality data were obtained from the Austrian Federal Statistical Agency. The date of the last follow-up was 1 March 2022.

### 2.4. Statistical Analysis

Continuous variables with non-normal distributions are described by the median and interquartile range (IQR) (25th and 75th percentiles). Total numbers and percentages were reported for categorical variables. Continuous variables with non-normal distributions were compared using the Mann-Whitney U test. Categorical variables were assessed using the chi-square test. The study employed a cohort design, utilizing individuals as the unit of analysis and the type of valve prosthesis (mechanical vs. biological) as the exposure variable. Within the study cohort, the age distribution displayed a bimodal pattern, with a pronounced decline observed at the age of 55. Based on the likelihood ratio test, no interaction was found for age. The multivariable Cox proportional hazards regression analysis was used to assess the effect of the prosthesis type on both the primary (composite outcome) and secondary study outcomes (individual components of the composite outcome). The effects were reported as an adjusted hazard ratio (HR) with a 95% confidence interval (CI). The co-variables that were likely to constitute common causes for the selection of valve prosthesis and each outcome were included in the multivariable models. The Kaplan-Meier curves were generated to visualize the cumulative probability of a composite outcome for both study groups. The multivariable logistic regression model was used to analyze the long-term neurological impairment during follow-up. A significance level of <0.05 was used to determine the statistical significance. Statistical analyses were performed using STATA 16.1 software (StataCorp LLC, College Station, TX, USA) and SPSS software 27.0 (IBM Corp, Armonk, NY, USA).

## 3. Results

Baseline characteristics

Among our study cohort, 34 patients (35.8%) received mechanical valve prostheses, whereas 61 patients (64.2%) received biological valve prostheses (Table 1). Recipients of mechanical valve prostheses were younger (median 47.4, IQR 41.6–56.7 vs. median 65.1, IQR 55.3–71.5; *p* < 0.001) and had a lower rate of hypertension (41.2% vs. 68.9%; *p* = 0.009) and diabetes mellitus (8.8% vs. 29.5%; *p* = 0.020). A higher rate of echocardiographic confirmed vegetation was observed in recipients of mechanical valve prostheses (100% vs. 85.3%; *p* = 0.019).

Detected pathogens in the blood culture

The species encountered in blood culture are summarized in Supplemental Table S1. For either prosthesis type, no significant difference was observed in the rates of pathogens in blood culture at baseline.

Neurological and radiological characteristics

Neuroimaging scans confirmed ischemic stroke as the predominant type of cerebral lesion in both study groups (Table 2). Only one patient presenting with pure hemorrhagic stroke at baseline received a mechanical valve prosthesis. The percentage of mixed lesions, comprising both ischemic and hemorrhagic stroke, was 17.7% and 13.1% in recipients of mechanical and biological valve prostheses, respectively. For either prosthesis type, no significant difference in the neurologic presentation at admission was observed. Time from stroke onset to surgery was similar between both groups. In terms of brain areas affected, middle cerebral artery involvement was similar in both groups. In contrast, recipients of mechanical valve prostheses had a significantly lower rate of cerebellar lesions (0% vs. 13.1%; *p* = 0.027).

Operative characteristics

The operative characteristics are provided in Table 3. Concerning urgency status, no differences were observed among the study groups. Aortic valve replacement (AVR), mitral valve replacement (MVR), and combined valve replacement were performed at similar rates. Regarding combined valve replacement, all patients received either mechanical or biological valve prostheses. Recipients of mechanical valve prostheses underwent aortic root enlargement more frequently (14.7% vs. 3.3%; *p* = 0.041). All other operative characteristics variables were comparable between study groups.

Postoperative in-hospital adverse events

For either prosthesis type, no significant difference was observed in the rates of postoperative in-hospital adverse events (Supplemental Table S2).

Antithrombotic therapy

Information regarding antithrombotic therapy, including anticoagulation and antiplatelet drugs, was collected upon discharge (Supplemental Table S3). Vitamin K antagonists were used more frequently in recipients of mechanical valve prostheses (35.3% vs. 8.2%; *p* ≤ 0.001). However, indirect thrombin inhibitors were used at a lower rate in this group (38.2% vs. 60.7%; *p* = 0.036). Regarding antiplatelet drugs, no difference was observed among the study groups. Long-term anticoagulation was typically switched to Vitamin K antagonists after discharge in patients with mechanical valves.

Primary study outcome—Composite outcome

**Figure 1:**
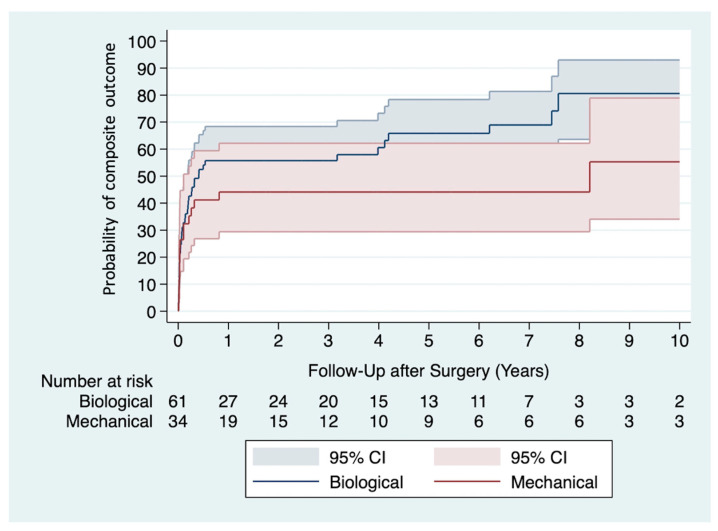
Kaplan-Meier curves show the probability of experiencing a composite outcome in recipients of mechanical versus biological valve prostheses.

A composite outcome was observed in 57 (60%) patients during the follow-up period. Multivariable Cox proportional hazards analysis showed a significantly lower risk of experiencing a composite outcome among recipients of mechanical valve prostheses (adjusted HR 0.46, 95% CI 0.22–0.96, *p* = 0.037) (Table 4). The Kaplan-Meier curves of both groups are depicted in Figure 1.

Secondary study outcomes

Analyses of individual components of the composite outcome showed that implantation of a mechanical valve prosthesis was not associated with an increased risk of ischemic stroke (adjusted HR 0.74, 95% CI 0.24–2.24, *p* = 0.590), hemorrhagic stroke (adjusted HR 0.31, 95% CI, 0.08–1.11; *p* = 0.073), and all-cause mortality (adjusted HR 0.48, 95% CI 0.21–1.09, *p* = 0.081) during the follow-up period (Table 5). Further, the risk of re-endocarditis was significantly lower in recipients of a mechanical valve prosthesis (adjusted HR 0.15, 95% CI 0.06–0.77, *p* = 0.026). Notably, a trend toward decreased risk of mRS deterioration throughout the follow-up period was observed in this group (adjusted odds ratio (OR) 0.22, 95% CI 0.05–1.02, *p* = 0.053) (Table 5).

Causes of death

The causes of death were comparable between study groups (Table 6).

## 4. Discussion

The main findings of the present study are as follows: (1) implantation of a mechanical valve prosthesis seems to be associated with a significantly lower risk of experiencing a composite outcome during the follow-up period; (2) implantation of a mechanical valve prosthesis was not associated with an increased risk of ischemic stroke, hemorrhagic stroke, or all-cause mortality during the follow-up period; (3) implantation of a mechanical valve prosthesis was associated with a significantly lower risk of re-endocarditis; and (4) a trend toward a lower risk of mRS deterioration during follow-up, which might be observed in recipients of mechanical valve prostheses.

To our knowledge, this is the first cohort study to compare the long-term clinical outcomes of recipients of mechanical versus biological valve prostheses for IE complicated by stroke.

The optimal timing of surgery after the onset of stroke is an important consideration in this clinical setting, involving a delicate balance between the potential neurological exacerbation and the risks associated with delaying the operation. The existing literature indicates that early surgery is advisable for patients with ischemic stroke, particularly in circumstances of high embolic risk, heart failure, and uncontrolled infection [16,17,18,19]. For individuals with hemorrhagic stroke, surgery within two weeks of stroke onset may offer benefits [20,21,22], while a few studies have indicated advantages in delaying surgery for more than one month [23]. In the present study, the time from stroke onset to surgery was not statistically significant between recipients of mechanical valve prostheses (median 13.5, IQR 3.4–29.4) and recipients of biological valve prostheses (median 10.0, IQR 5.8–17.7), and *p* = 0.470.

Implantation of mechanical valve prostheses in patients presenting with stroke at baseline remains a contentious issue due to the conceivable risk of stroke exacerbation attributed to anticoagulant therapy [6,7]. In contrast, mandatory anticoagulant therapy, along with the implantation of a mechanical valve prosthesis, might potentially provide protection against new-onset stroke during the follow-up period [8,10,11]. Our analysis suggests that the implantation of a mechanical valve prosthesis was not associated with an increased risk of ischemic stroke or hemorrhagic stroke during the follow-up period. Nevertheless, in elective clinical settings, several studies have reported that recipients of mechanical prostheses are at a higher risk of new-onset bleeding yet a lower risk of reoperation during the follow-up period compared to those of biological valve prostheses [24,25]. Offering a non-mechanical valve substitute might be beneficial in certain patient groups such as women of childbearing age, individuals engaged in physically demanding work, and those with a high likelihood of requiring extended mechanical circulatory support [3,26,27].

Indeed, the long-term neurological outcome is undoubtedly a crucial aspect to be considered in patients with stroke at baseline undergoing surgery for IE. According to the current analysis, recipients of mechanical valve prostheses exhibited a trend toward a lower risk of mRS deterioration during the follow-up period (adjusted OR 0.22, 95% CI 0.05–1.02, *p* = 0.053). Notably, patients treated with anticoagulant therapy may have long-term clinical benefits in terms of stroke prophylaxis and improved neurological recovery [8,9,28]. The literature reports complete neurological recovery (mRS = 0) in 70% of patients undergoing surgery for IE complicated by stroke (irrespective of the type of valve prosthesis) [19]. In a similar manner, Murai and associates documented complete neurological recovery in patients who underwent early surgery for IE [29]. Nevertheless, in the present study, the recipients of mechanical valve prostheses were younger and potentially had a higher neurological recovery potential.

Concerning mortality, numerous studies have examined the impact of hemorrhagic and ischemic strokes at baseline on postoperative mortality in patients undergoing surgery for IE, regardless of the type of valve prosthesis. However, these studies have yielded diverse findings. Several analyses have indicated increased mortality in patients undergoing surgery for IE complicated by stroke [19,29]. Conversely, a study by Diab and colleagues found that preoperative intracranial hemorrhage was not associated with increased in-hospital mortality [30]. Likewise, a large retrospective analysis by Said et al. concluded that stroke prior to surgery for IE was not associated with increased mortality [31]. Nevertheless, several analyses have investigated preferential types of valve prostheses in the context of IE and reported that implantation of a mechanical valve prosthesis was not associated with an increased risk of all-cause mortality [32,33], which is in line with our data.

The present study suggests a lower rate of experiencing a composite outcome in recipients of mechanical valve prostheses. This finding appears to be primarily driven by a significantly decreased risk of re-endocarditis among recipients of mechanical valve prostheses (Table 5). Notably, similar results have been reported in the literature, reinforcing the potential benefits of mechanical valve prostheses in terms of a lower risk of re-endocarditis [34,35,36,37,38].

Regarding the main indications for surgery, no significant differences in the rates of heart failure, uncontrolled infection, and high embolic risk were found between recipients of mechanical and biological valve prostheses. Pizzino et al. conducted a study analyzing the composite outcomes of all-cause mortality, hospitalizations, and relapses of IE among patients categorized by the primary indication for surgery. However, the findings revealed that patients presenting with heart failure and an elevated risk of embolism were independently associated with a higher risk of experiencing a composite outcome [39].

Nonetheless, it is essential to highlight the critical role of a multidisciplinary endocarditis team in evaluating the risk-benefit assessment for each patient and clinical situation [4]. Postoperative close monitoring of the coagulation profile is indispensable in this clinical setting [10].

## 5. Study Strengths and Limitations

The findings of this study may provide valuable insights into this specific clinical setting and serve as a basis for future research. Our study has the inherent limitations of a retrospective single-center analysis. Most importantly, the size of our study cohort is small. Additionally, the time interval from stroke to surgery was heterogeneous and was defined within a 2 months timeframe. A detailed analysis of the extent of the infarcted area was not performed. The mRS scores at baseline were assessed retrospectively. The study groups differed in sample size and baseline characteristics such as hypertension and diabetes mellitus. Molecular analyses, including the examination of Cluster of Differentiation 93 gene dysregulation and its potential association with cardiovascular outcomes, have not been investigated [40]. Moreover, information regarding adherence to anticoagulant therapy at baseline and during the follow-up period was not available for the statistical analysis.

## 6. Conclusions

In conclusion, the implantation of mechanical valve prostheses in patients presenting with IE complicated by stroke might be beneficial in terms of a reduced the risk of experiencing the composite outcome and demonstrating similar risks of ischemic stroke, hemorrhagic stroke, and all-cause mortality during the follow-up period compared with biological valve prostheses. Further studies would be needed to validate our results.

## Figures and Tables

**Table 1:** Baseline characteristics.

Variables	Mechanical *n* = 34 (35.8%)	Biological *n* = 61 (64.2%)	*p*-Value
Age (years) (25th–75th interval)	47.4 (41.6–56.7)	65.1 (55.3–71.5)	<0.001
Age > 55 years (%)	10 (29.4)	45 (73.8)	<0.001
Female (%)	10 (29.4)	20 (32.8)	0.734
EuroSCORE II (25th–75th interval)	7.5 (2.4–18.1)	10.3 (5.2–24.6)	0.073
Ejection fraction > 60% (%)	27 (79.4)	49 (80.3)	0.915
Atrial fibrillation (%)	10 (29.4)	14 (23.0)	0.487
Hypertension (%)	14 (41.2)	42 (68.9)	0.009
Diabetes mellitus (%)	3 (8.8)	18 (29.5)	0.020
Dialysis (%)	2 (5.9)	7 (11.5)	0.372
Prosthetic valve endocarditis (%)	6 (17.7)	14 (23.0)	0.543
Positive blood culture (%)	32 (94.1)	59 (96.7)	0.545
Laboratory data (25th–75th interval)			
C-reactive protein (mg/dL)	6.0 (2.9–9.0)	6.7 (3.8–14.1)	0.244
White blood cell count (G/L)	8.5 (6.7–12.4)	9.0 (7.1–12.7)	0.650
Lactate value (mmol/L)	1.0 (0.7–1.5)	1.0 (0.8–1.3)	0.941
Positive echocardiography findings			
Vegetation (%)	34 (100)	52 (85.3)	0.019
Annular abscess (%)	8 (23.5)	17 (27.9)	0.645
Pseudoaneurysm (%)	2 (5.9)	1 (1.6)	0.257
Fistula (%)	1 (2.9)	1 (1.6)	0.672
Peripheral embolism (%)	16 (47.1)	37 (60.7)	0.201
Main indication for surgery			
Heart failure (%)	14 (41.2)	16 (26.2)	0.133
Uncontrolled infection (%)	10 (29.4)	23 (37.7)	0.416
High embolic risk (%)	10 (29.4)	22 (36.1)	0.511
Preoperative antibiotic treatment (d) (25th–75th interval)	14.0 (6.6–24.8)	10.3 (6.4–22.4)	0.342

Boldface denotes statistical significance (*p* < 0.05). Euro SCORE II: European System for Cardiac Operative Risk Evaluation II.

**Table 2:** Neurological and radiological characteristics.

Variables	Mechanical *n* = 34 (35.8%)	Biological *n* = 61 (64.2%)	*p*-Value
Stroke prior to surgery			
Ischemic (%)	27 (79.4)	49 (80.3)	0.915
Hemorrhagic (%)	1 (2.9)	4 (6.6)	0.449
Mixed lesion (%)	6 (17.7)	8 (13.1)	0.550
Time from stroke to surgery (d) (25th–75th interval)	13.5 (3.4–29.4)	10.0 (5.8–17.7)	0.470
Neurologic presentation at admission			
mRS score ≥ 4 (%)	16 (47.1)	29 (47.5)	0.964
Motor control impairment (%)	14 (41.2)	28 (45.9)	0.657
Impaired consciousness (%)	4 (11.8)	17 (27.9)	0.070
Asymptomatic (%)	7 (20.6)	6 (9.8)	0.144
Brain area affected			
Middle cerebral artery (%)	11 (32.4)	13 (21.3)	0.235
Multiple site (%)	12 (35.3)	20 (32.8)	0.804
Frontoparietal (%)	7 (20.6)	12 (19.7)	0.915
Temporal (%)	3 (8.8)	5 (8.2)	0.916
Occipital (%)	4 (11.8)	7 (11.5)	0.966
Cerebellum (%)	0 (0.0)	8 (13.1)	0.027
Brain Stem (%)	2 (5.9)	1 (1.6)	0.257
Basal ganglia (%)	1 (2.9)	9 (14.8)	0.072

Boldface denotes statistical significance (*p* < 0.05). mRS: modified Rankin scale.

**Table 3:** Operative characteristics.

Variables	Mechanical *n* = 34 (35.8%)	Biological *n* = 61 (64.2%)	*p*-Value
Urgency status			
Urgent (%)	28 (82.4)	46 (75.4)	0.434
Emergent (%)	6 (17.6)	13 (21.3)	0.669
Salvage (%)	0 (0.0)	2 (3.3)	0.056
AVR (%)	12 (35.3)	25 (41.0)	0.586
MVR (%)	18 (52.9)	26 (42.6)	0.334
Combined AVR and MVR (%)	4 (11.8)	10 (16.4)	0.542
Right side involvement (%)	2 (5.9)	3 (4.9)	0.840
CABG (%)	3 (8.8)	12 (19.7)	0.164
Annular abscess exclusion (%)	6 (17.6)	18 (29.5)	0.202
Commando procedure (%)	2 (5.9)	0 (0.0)	0.056
Ventricular septal defect closure (%)	1 (2.9)	1 (1.6)	0.672
Aortic root enlargement (%)	5 (14.7)	2 (3.3)	0.041
Aortic root replacement (%)	4 (11.8)	2 (3.3)	0.103
CPB time (min) (25th–75th interval)	148 (95–221)	145 (108–192)	0.951
Cross-clamp time (min) (25th–75th interval)	103 (63–164)	103 (71–129)	0.816

Boldface denotes statistical significance (*p* < 0.05). AVR: aortic valve replacement; CABG: coronary artery bypass grafting; CPB: cardiopulmonary bypass; MVR: mitral valve replacement.

**Table 4:** Primary study outcome—Composite outcome.

Variables	HR	95% CI	*p*-Value
Mechanical valve prosthesis ^a^	0.46	0.22–0.96	0.037
Age > 55 years	0.42	0.19–0.94	0.035
EuroSCORE II (log-transformed)	1.49	1.07–2.06	0.017
Time from stroke to surgery (days)	0.98	0.96–1.00	0.078
Atrial fibrillation	1.74	0.88–3.46	0.112
Intravenous drug abuse	0.99	0.40–2.43	0.984
Prosthetic valve endocarditis	0.82	0.40–1.67	0.579
Annular abscess	1.32	0.69–2.52	0.398
Cross-clamp time (min)	1.00	1.00–1.01	0.417

^a^ Effects presented as HR using multivariable Cox proportional hazards regression analysis. Boldface denotes statistical significance (*p* < 0.05). CI: confidence interval; EuroSCORE II: European System for Cardiac Operative Risk Evaluation II; HR: hazard ratio.

**Table 5:** Secondary study outcomes.

Variables	Multivariable Relative Effects	*p*-Value
**Ischemic stroke** ^a^		
Mechanical valve prosthesis	0.74 (0.24–2.24)	0.590
**Hemorrhagic stroke** ^a^		
Mechanical valve prosthesis	0.31 (0.08–1.11)	0.073
**Re-endocarditis** ^a^		
Mechanical valve prosthesis	0.15 (0.06–0.77)	0.026
**All-cause mortality** ^a^		
Mechanical valve prosthesis	0.48 (0.21–1.09)	0.081
**mRS deterioration during follow-up** ^b^		
Mechanical valve prosthesis	0.22 (0.05–1.02)	0.053

^a^ Effects presented as HR and 95% CI using multivariable Cox proportional hazards regression analysis. ^b^ Effects presented as OR and 95% CI using multivariable logistic regression analysis. Co-variables for these analyses were age > 55 years, EuroSCORE II (log-transformed), time from stroke to surgery (days), atrial fibrillation, intravenous drug abuse, prosthetic valve endocarditis, annular abscess, and cross-clamp time (min). Boldface denotes statistical significance (*p* < 0.05). CI: confidence interval; mRS: modified Rankin scale.

**Table 6:** Causes of death.

Variables	Mechanical *n* = 34 (35.8%)	Biological *n* = 61 (64.2%)	*p*-Value
Cardiac (%)	4 (11.8)	7 (11.5)	0.966
Infection (%)	3 (8.8)	10 (16.4)	0.303
Multi-organ failure (%)	2 (5.9)	3 (4.9)	0.840
Neurological (%)	1 (2.9)	4 (6.6)	0.449
Cancer (%)	0 (0.0)	3 (4.9)	0.286
Other (%)	1 (2.9)	3 (4.9)	0.647

## Data Availability

The raw data supporting the conclusions of this article will be made available by the authors upon request.

## Supplementary Materials

The following supporting information can be downloaded at https://www.mdpi.com/article/10.3390/jcm13195712/s1, Table S1: Detected pathogens in the blood culture; Table S2: Postoperative in-hospital adverse events; Table S3: Antithrombotic therapy.

## Abbreviations

AVR	Aortic valve replacement
CABG	Coronary artery bypass grafting
CI	Confidence interval
CPB	Cardiopulmonary bypass
ECMO	Extracorporeal Membrane Oxygenation
Euro SCORE II	European System for Cardiac Operative Risk Evaluation II
HR	Hazard ratio
IE	Infective endocarditis
IQR	Interquartile range
mRS	modified Rankin Scale
MVR	Mitral valve replacement
OR	Odds Ratio
