# Supplementary material for: Mechanical versus Biological Valve Prostheses for Infective Endocarditis Presenting with Stroke
Source: J Clin Med. 2024 Sep 25;13(19):5712. doi: 10.3390/jcm13195712 (PMC11477093; doi:10.3390/jcm13195712)
Supplement: Supplementary file 1 [file jcm-13-05712-s001.zip › jcm-3201564-supplementary.pdf]

# Supplementary Material

**Supplemental Table S1: Detected pathogens in the blood culture**

| Variables | Mechanical N = 34 (35.8%) | Biological N = 61 (64.2%) | p-value |
|---|---|---|---|
| Staphylococcus aureus (%) | 14 (41.2) | 22 (36.1) | 0.623 |
| Viridans group streptococci (%) | 7 (20.6) | 8 (13.1) | 0.338 |
| Coagulase-negative staphylococci (%) | 4 (11.8) | 4 (6.6) | 0.381 |
| Enterobacteriaceae (%) | 1 (2.9) | 3 (4.9) | 0.646 |
| Enterococcus faecium (%) | 2 (5.9) | 5 (8.2) | 0.679 |
| Group B Streptococci (%) | 0 (0.0) | 2 (3.3) | 0.286 |
| Other (N = 1 each) (%) | 2 (5.9) | 5 (8.2) | 0.679 |
| Multiple species (> 1 species) (%) | 2 (5.9) | 10 (16.4) | 0.139 |

Values are presented as n (%).
Categorical variables were compared using chi-square test.

**Supplemental Table S2: Postoperative in-hospital adverse events**

| Variables | Mechanical N = 34 (35.8%) | Biological N = 61 (64.2%) | p-value |
|---|---|---|---|
| New-onset atrial fibrillation (%) | 8 (23.5) | 9 (14.8) | 0.285 |
| Dialysis (%) | 5 (14.7) | 13 (21.3) | 0.431 |
| ECMO-support (%) | 9 (26.5) | 8 (13.1) | 0.104 |
| Prolonged intubation time ≥ 48h (%) | 15 (44.1) | 30 (49.2) | 0.636 |
| Prolonged intensive care unit stay ≥ 7days (%) | 17 (50.0) | 34 (55.7) | 0.591 |
| 30-day mortality (%) | 5 (14.7) | 3 (4.9) | 0.100 |

Values are presented as n (%).
Categorical variables were compared using chi-square test.
ECMO: Extra-corporeal membrane oxygenation.

**Supplemental Table S3: Antithrombotic therapy**

| Variables | Mechanical N = 34 (35.8%) | Biological N = 61 (64.2%) | p-value |
|---|---|---|---|
| Anticoagulation drugs (%) | 25 (73.5) | 42 (68.9) | 0.632 |
| Vitamin K antagonist | 12 (35.3) | 5 (8.2) | **<0.001** |
| Indirect thrombin inhibitor | 13 (38.2) | 37 (60.7) | **0.036** |
| Antiplatelet drugs (%) | 9 (26.5) | 25 (41.0) | 0.157 |
| Acetylsalicylic acid | 8 (23.5) | 25 (41.0) | 0.087 |
| Clopidogrel | 1 (2.9) | 1 (1.6) | 0.672 |

Values are presented as n (%).
Categorical variables were compared using chi-square test.
